# Safe Implementation of Robotic Colorectal Surgery: Balancing Surgical Training and Patient Safety with the Dual-Console System

**DOI:** 10.3390/jcm15145669

**Published:** 2026-07-20

**Authors:** Jurij Aleš Košir, Miha Petrič, Blaž Trotovšek, Gregor Norčič, Jan Grosek

**Affiliations:** 1 Department of Abdominal Surgery, University Medical Centre Ljubljana, Zaloška 7, 1000 Ljubljana, Slovenia; miha.petric@kclj.si (M.P.);; 2 Faculty of Medicine, University of Ljubljana, Korytkova 2, 1000 Ljubljana, Slovenia

**Keywords:** robotic colorectal surgery, learning curve, safe implementation, complications

## Abstract

**Background**: Robotic platforms expand minimally invasive options in colorectal surgery but raise concerns about training and patient safety. Dual-console systems may enable real-time coaching while preserving outcomes. **Methods**: We implemented a structured framework for safe implementation of robotic colorectal surgery that balances trainee autonomy with patient safety using a dual-console model. Early cases emphasized low-complexity pathology with escalation by predefined benchmarks. We prospectively gathered data and evaluated early program outcomes with primary endpoints including intraoperative adverse events, conversion to open surgery, 30-day morbidity, anastomotic integrity, and oncologic quality metrics. Secondary endpoints included operative time. **Results**: We included the analysis of 17 patients operated by one surgeon under supervision and compared the results to other senior colorectal surgeons. Out of the 17 patients, there were no conversions or anastomotic leaks. Overall complications were comparable to baseline robotic cases. Resection margins and lymph node yields met oncologic standards. Operative times were longer during early adoption but approached baseline with progression. Operative times were significantly reduced after eight cases and approached the times of senior surgeons after 13 cases. **Conclusions**: A dual-console strategy enables safe, scalable training in robotic colorectal surgery without compromising short-term patient outcomes or oncologic quality. Key elements include rigorous case selection, proficiency-based progression, real-time coaching, standardized protocols, and continuous data surveillance. This framework can guide institutions seeking to expand robotic colorectal programs while safeguarding patients and accelerating the learning curve.

## 1. Introduction

The adoption of robotic-assisted surgery (RAS) in colorectal procedures has grown substantially since the introduction of new systems over the last 15 years [1,2]. RAS offers advantages over conventional laparoscopy, including enhanced three-dimensional visualization, improved dexterity, tremor filtration, and superior ergonomics for the surgeon [3]. These features are particularly beneficial in complex pelvic dissections, such as total mesorectal excision (TME) for rectal cancer, where precise dissection in confined spaces is critical to preserve autonomic nerves, achieve clear margins, and minimize complications [4].

Despite these benefits, the integration of RAS into colorectal practice introduces challenges. The learning curve for robotic colorectal surgery is well-documented, with studies estimating 15–50 cases are required to achieve proficiency, depending on prior laparoscopic experience and procedure complexity [5,6]. During this phase, risks include prolonged operative times, higher conversion rates to open surgery, and potential increases in complications if training is unstructured [7].

Patient safety remains paramount [8]. Historical concerns with new technologies—such as early laparoscopic cholecystectomy series—highlight the need for deliberate, evidence-based implementation. In robotic surgery, the dual-console configuration of modern platforms represents a major advance [9]. It allows a supervising surgeon (proctor) and trainee to share control in real time: the proctor can intervene instantly via “swap” or “give-and-take” functions, provide guidance on camera control or accessory arms, and reduce trainee stress while enabling progressive autonomy [10].

At the University Medical Centre Ljubljana (UMCL), robotic colorectal surgery was introduced as part of a broader minimally invasive program. Building on institutional experience with robotic pancreatic and other abdominal procedures, we implemented a structured dual-console framework to train surgeons safely [6,10,11]. This report describes our early experience with 17 supervised cases performed by a single trainee surgeon, comparing outcomes to established senior robotic colorectal surgeons at our center. Our aim was to demonstrate that intentional use of the dual console, combined with case selection and benchmark progression, preserves short-term safety and oncologic quality during the adoption phase [12].

## 2. Materials and Methods

### 2.1. Study Design and Setting

This is an analysis of prospectively collected data from the initial phase of our robotic colorectal surgery program at UMCL, a tertiary academic center. The robotic colorectal program launched in 2020, following certification of surgeons on the da Vinci Xi system, which includes a dual-console capability [1]. All of the operations were performed from early 2025 to the beginning of 2026, overlapping with the period the trainee performed operations on the robotic system.

### 2.2. Training Framework

We adopted a proficiency-based, stepwise training model emphasizing dual-console utilization.

Phase 1 (preparation): Simulator training (da Vinci Skills Simulator) targeting console proficiency, followed by observation and assisting of ≥100 robotic colorectal cases. The trainee also visited an expert learning center at another institution (Robotic Academy Intuitive Naples, Naples, Italy) and also participated in a 3-month fellowship at an expert robotic surgery center (Meander Medisch Centrum, Amersfoort, The Netherlands). He also had experience with performing about 30 laparoscopic colorectal resections before starting on the robotic platform.

Phase 2 (supervised introduction): Trainee operates under direct proctoring on the dual console for low-complexity cases (e.g., right hemicolectomy for benign or early-stage malignancy, sigmoid resection).

Phase 3 (progressive autonomy): Escalation to moderate/high-complexity procedures (e.g., large tumors, low anterior resection, TME) based on predefined benchmarks: no intraoperative adverse events in prior five cases, operative time within 20% of proctor baseline, R0 margins, ≥12 lymph nodes harvested.

Real-time coaching: Proctor is present behind the second console ready to intervene. With increasing experience, the trainee performs cases without supervision [9].

Case selection: Early cases excluded emergencies, T4 tumors, prior extensive abdominal surgery, or severe obesity (BMI > 35 kg/m^2^).

Standardized protocols: Enhanced recovery after surgery pathway, uniform port placement, vessel ligation technique, intracorporeal anastomosis where applicable, and leak testing.

### 2.3. Patients

We analyzed the first 17 consecutive robotic colorectal resections performed by one trainee surgeon (J.A.K.) under supervision; operations were performed from early 2024 to the beginning of 2026. Inclusion: elective curative-intent resections for colorectal pathology performed on the robotic system. Exclusion: hybrid laparoscopic-robotic cases or non-colorectal procedures.

Outcomes were benchmarked against 34 concurrent robotic colorectal cases performed by two senior surgeons (J.G., G.N.; experienced in > 100 robotic cases each) during the same period as a case–control study. The comparison group met the same inclusion criteria as for the case selection mentioned above. The cases were not consecutive and were chosen for analysis from a larger pool of patients on whom the two experienced surgeons operated. The cases for comparison were picked to match the trainee cases in the following characteristics: age, sex, BMI and ASA score. Complications were graded as severe if they were classified as Clavien–Dindo IIIb or more.

### 2.4. Endpoints

Primary:

Intraoperative adverse events (unintended organ injury, major bleeding > 500 mL);
Conversion to open or hand-assisted laparoscopy;
Thirty-day morbidity (Clavien–Dindo classification);
Anastomotic leak (clinical/radiologic);
Oncologic metrics (R0 resection, lymph node yield).

Secondary: operative time (skin-to-skin; time from incision to closure of wounds), console time, length of stay (LOS).

Data were extracted from electronic health records and our robotic surgery registry. Data were prospectively collected including operative times, adverse events, oncologic metrics, patient characteristics, readmission rate and length of stay. We analyzed the operative times of the trainee surgeon and compared them to operative times of the two senior surgeons in chronological order. Statistical comparison used descriptive statistics, *t*-tests for continuous variables, and chi-square/Fisher’s exact for categorical variables (significance *p* < 0.05). A *p*-value of less than 0.05 was considered statistically significant and the analysis was performed using SPSS version 20 for Windows. During the preparation of this manuscript, the authors used ChatGPT-4 for the purposes of language refinement and improving readability. After using this tool, the authors reviewed and edited the output and take full responsibility for the content of this publication.

## 3. Results

### 3.1. Patient Characteristics

The trainee series (*n* = 17) included 10 right hemicolectomies, five sigmoid resections, and two rectal resections (low anterior) (Table 1). Mean age was 68.4 years (SD 9.2), 59% were male, and mean BMI was 27.1 kg/m^2^ (SD 4.3). Indications: 12 colon cancers (stages I–III), two rectal cancers, two diverticulosis and one Crohn’s disease. ASA score distribution: II (65%), III (35%).

The comparison group (*n* = 34) had similar demographics (mean age 67.8, BMI 26.8, 56% male) and case mix (proportionally more rectal cases). There were similar numbers of ASA II and III patients in each group.

### 3.2. Perioperative Outcomes

No intraoperative adverse events occurred in the trainee series (Table 2). There were zero conversions to open surgery. In comparison, senior surgeons had one anastomotic leak and zero conversions (consistent with low conversion rates in structured programs) [13].

There was one anastomotic leak in the control group. Thirty-day complications (any Clavien–Dindo): trainee 23.5% (4/17; all four grades I–II); senior surgeons 26.5% (9/34; including one Clavien–Dindo IIIb for anastomotic leak). No grade ≥ IIIb complications or mortality in trainee cases (aligning with safety in dual-console series) [14,15].

Regarding oncologic quality, all resections were R0. Median lymph nodes: 16 (range 12–24) in trainee vs. 17 (13–32) in senior surgeons (*p* = 0.62). For rectal cases (*n* = 2 trainee), CRM > 1 mm in all (meeting standards reported in recent learning curve analyses) [5,16].

**Figure 1:**
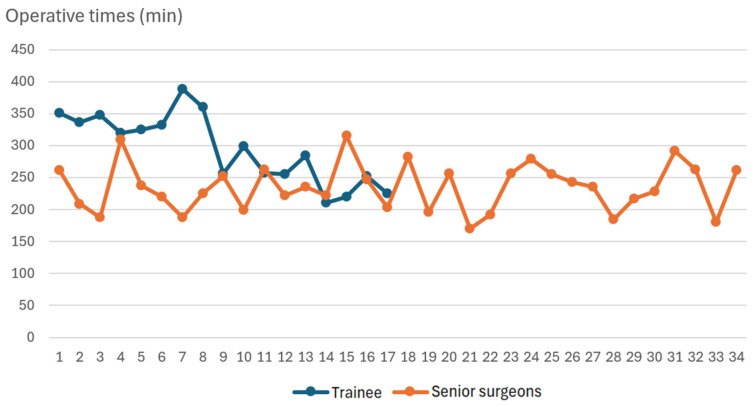
Operative times (skin–skin) of the trainee and senior surgeons.

Mean operative time for the trainee was 295 min (SD 52) vs. senior surgeons 234 min (SD 36) (*p* = 0.008). Console time: trainee 265 min vs. senior surgeons 210 min. Early cases (first eight) averaged 345 min; later cases (9–17) 251 min, approaching senior-surgeon baseline (reflecting typical progression in structured dual-console training) (Figure 1) [6,15,17]. The senior surgeons were operating at stable operating times throughout the study period.

## 4. Discussion

Our early experience confirms that a structured dual-console approach enables safe introduction of robotic colorectal surgery during the learning phase [9,10]. Key findings—no conversions, no leaks, comparable morbidity, and preserved oncologic metrics—align with recent reports emphasizing immersive, supervised training [12,18].

The dual console addresses core training-safety tensions. Traditional proctoring (bedside guidance) limits real-time intervention; simulation alone lacks operative context. The dual console allows graduated responsibility: initial shared control builds confidence in wristed instruments and the three-handed technique, while proctor oversight mitigates errors [9]. The literature supports this: studies from structured programs show equivalent outcomes between trainees and consultants on the dual-console system, with no compromise in oncologic parameters or efficiency over time [10,19,20]. Similarly, modular TME training programs report no differences in R0 rates or lymph node yields [13].

Learning curves in robotic colorectal surgery vary. They can be studied by measuring operative times, complications, or oncological outcomes with the latter two being more important. However, many learning curves are based on operative times because of ease of analysis and the lower number of cases needed to show progression in skill. Right-sided procedures often plateau earlier (~15–25 cases) than rectal (~25–36+ cases) [5,6,21,22,23]. Our trainee, who had a strong laparoscopic background, showed time reduction after ~8 cases, consistent with studies factoring complications into curve analysis (e.g., significant drop of the curve after 13 cases) [7,15]. The trainee did not reach the plateau of the senior surgeons yet; however, he was approaching it after the first 13 cases.

Case selection was critical. Starting with low-complexity pathology minimized risk during skill acquisition. Benchmarks ensured readiness before escalation, preventing premature exposure to high-stakes dissections [11]. In our study we included low-risk patients and therefore the rate of complications was also low. This, along with the low sample size, contributed to the comparable rate of complications of both groups. The comparison group had more rectal cases, which have more complications than operations on the colon. The Clavien–Dindo IIIb complication could be attributed to this imbalance that is in favor of the trainee. Also, the imbalance may in the same manner influence the operative times of the experienced surgeons.

The longer time of operation for the trainee can be attributed mostly to longer console time, as the docking at our facility is usually done by the trainee at the bedside. Longer console time is due to more unnecessary movements and steps with additional time dedicated to teaching.

Limitations include the small sample size and single-trainee focus. Selection bias (easier cases early) may favor outcomes, though concurrent comparison with senior surgeons mitigates this. Long-term oncologic follow-up (e.g., 3–5-year recurrence) is pending.

## 5. Conclusions

Robotic colorectal surgery can be implemented safely via a dual-console framework that integrates rigorous selection, proficiency progression, real-time mentoring, standardization, and surveillance. In our initial 17 supervised cases, patient safety and oncologic quality matched experienced surgeons, despite longer early operative times that improved rapidly.

This model offers a blueprint for institutions establishing or expanding robotic programs, particularly in academic settings prioritizing training. As robotic platforms evolve, structured dual-console adoption will remain essential to balance innovation, education, and safety.

## Figures and Tables

**Table 1:** Patient demographics.

Variable	Trainee (*n* = 17)	Senior Surgeons (*n* = 34)
Mean age	68.4	67.8
Male	59%	56%
Mean BMI	27.1	26.8
ASA II	65%	68%
ASA III	35%	32%

**Table 2:** Operative outcomes.

Metric/Outcome	Trainee (*n* = 17)	Senior Surgeons (*n* = 34)	*p*-Value
Intraoperative adverse events	0 (0%)	0 (0%)	-
Conversions to open surgery	0 (0%)	0 (0%)	-
Median hospitalization (days)	6	6	-
Any complication (Clavien I–V)	4 (23.5%)—all I–II	9 (26.5%)—includes one IIIb	-
Mortality (30-day)	0 (0%)	0 (0%)	-
Readmission (30-day)	1 (6%)	3 (9%)	*p* = 0.43
R0 resection rate	100% (all)	100% (all)	-
Median lymph nodes (range)	16 (12–24)	17 (13–32)	*p* = 0.62
Operative time (skin-to-skin)	295 ± 52 min	234 ± 36 min	*p* < 0.001
Mean console time	265 min	210 min	*p* < 0.001
Mean operative time early cases (1–8)	345 min	-	-
Mean operative time later cases (9–17)	251 min	-	-

## Data Availability

The data that support the findings of this study are available from the corresponding author upon request.

## Abbreviations

The following abbreviations are used in this manuscript:

RAS	Robotic-assisted surgery
TME	Total mesorectal excision
UMCL	University Medical Center Ljubljana
CRM	Circumferential resection margin
BMI	Body mass index
LOS	Length of stay
